# Protective Role of Testicular Hormone INSL3 From Atrophy and Weakness in Skeletal Muscle

**DOI:** 10.3389/fendo.2018.00562

**Published:** 2018-09-28

**Authors:** Alberto Ferlin, Luca De Toni, Alexander I. Agoulnik, Giorgia Lunardon, Andrea Armani, Sergia Bortolanza, Bert Blaauw, Marco Sandri, Carlo Foresta

**Affiliations:** ^1^Unit of Endocrinology, Department of Clinical and Experimental Sciences, University of Brescia, Brescia, Italy; ^2^Department of Medicine, University of Padova, Padova, Italy; ^3^Department of Human and Molecular Genetics, Herbert Wertheim College of Medicine, Florida International University, Miami, FL, United States; ^4^Department of Biomedical Sciences, University of Padova, Padova, Italy; ^5^Venetian Institute of Molecular Medicine, Padova, Italy

**Keywords:** INSL3, Leydig cells, hypogonadism, leg denervation, protein synthesis, ubiquitin-proteasome system

## Abstract

Androgens are primarily involved in muscle growth, whilst disease-driven muscle wasting is frequently associated with hypogonadism. The Leydig cells of the testes also produce the peptide-hormone Insulin-like peptide 3 (INSL3). INSL3 displays anabolic activity on bone, a target tissue of androgens, and its plasma concentrations are diminished in male hypogonadism. Here we tested the role of INSL3 on muscle mass regulation, in physiological and pathological conditions. Studies on C2C12 cell line showed that INSL3, acting on his specific receptor RXFP2, promotes skeletal muscle protein synthesis through the Akt/mTOR/S6 pathway. Next, studies on *Rxfp2*^−/−^ mice showed that INSL3 is required to prevent excessive muscle loss after denervation. Mechanistically, denervated *Rxfp2*^−/−^ mice lacked the compensatory activation of the Akt/mTOR/S6 pathway and showed an abnormal ubiquitin-proteasome system activation. Lack of INSL3 activity resulted also in reduced contractile force. These findings underlie a role of INSL3/RXFP2 in protein turnover, contributing to muscle wasting in male hypogonadism.

## Introduction

The skeletal muscle mass is regulated by the coordinated balance between rates of protein synthesis and protein breakdown. The decrease in muscle mass and fiber size is associated with a number of clinical conditions, from aging and starvation to disuse, cancer and altered hormonal pattern (1, 2). Androgens represent the class of sex steroids with a major involvement in muscle mass growth (3, 4). Indeed, much of the focus on androgens and testosterone (T) dealt in particular with hypogonadal men since the disease-driven muscle catabolism frequently correlates with reduced circulating T levels (5). From a clinical point of view, reduced muscle mass and strength represent major manifestations that raise suspicion of T deficiency (6). In animal models, T loss in male mice decreases muscle Igf1 mRNA, Akt phosphorylation and the rate of myofibrillar protein synthesis and these changes are all reversed by androgen treatment (3).

Tastosterone has been classically considered the major connection between testis function and skeletal muscle, as well as other target tissues such as bone. However, the endocrine activity of the testis has been recently widened to other non-steroidal hormones that are produced by Leydig cells and elicit anabolic action, in particular Insulin-like peptide 3 (INSL3) [reviewed in (7)]. INSL3, by acting on gubernaculum, has a major role in testis descent during fetal development (8, 9) whereas its role in adulthood has not been fully understood (7, 8, 10). In adults, INSL3 is constitutively produced by the Leydig cells since the achievement of full cell differentiation. In addition, marked luteinizing hormone (LH)-induced up-regulation of *INSL3* gene expression has been largely documented (11–13). On these bases, variations in serum levels of INSL3 are more reliable marker of Leydig cell function compared to T [reviewed in (7, 10)]. Low levels of circulating INSL3 are evident during hypogonadism, male infertility, and normal aging (14), but little is known about possible phenotypes associated with this low INSL3 concentrations. Indeed, the INSL3 specific receptor RXFP2 is expressed in many tissues besides the gubernaculum, including kidney, thyroid, pituitary gland, brain, bone, and skeletal muscle (8, 10–12, 15–17). Importantly, we showed that young men with the T222P inactivating mutation in the *RXFP2* gene and *Rxfp2*^−/−^ mice have significantly reduced bone mineral density and increased risk of osteoporosis (17). INSL3 has an overall anabolic action on bone, mainly acting on bone forming cells, the osteoblasts (17), similarly to what observed for T.

Since skeletal muscle and bone form a functional unit and share different hormones with anabolic action such as T, Insulin, BMPs (18), we tested the role of INSL3/RXFP2 signaling pathway on muscle mass maintenance in physiological and pathological conditions.

## Materials and methods

### Cell cultures

C2C12 mouse myoblast cell line was purchased from ATCC® (Manassas, USA). Cells were cultured in DMEM (Gibco®), supplemented with 10% fetal bovine serum and 100 μg/ml penicillin/streptomycin (growth medium), at 37°C in a 5% CO2 incubator. Differentiation into myotubes was induced by the use of DMEM supplemented with 2% horse serum. Mouse INSL3 (Phoenix Pharmaceuticals, Burlingame, CA, USA) and INSL3-β chain dimer (β-chain) were used at the indicated concentration in stimulation experiments, equally performed at 37°C in a 5% CO2 incubator. In differentiation experiments, culture medium containing INSL3, and β-chain when necessary, was renewed every other day. INSL3-β chain is an antagonist of RXFP2 receptor (19) and it was provided by Prof. Ross Bathgate (Florey Institute of Neuroscience and Mental Health, The University of Melbourne, Victoria, Australia).

The effect of INSL3 on C2C12 myblasts proliferation was evaluated by MTT assay as previously described (20).

Immunofluorescence was performed on cells fixed with paraformaldehyde 4% in PBS, permeabilized with Triton 1% and saturated with normal donkey serum (NDA) 5%-in bovine serum albumin (BSA) solution. Primary antibodies, rabbit anti-RXFP2 (21) (aa 144-158, cat# ABIN615664, Antibodies Online, Atlanta, GA, USA; dilution 1:1000) and mouse anti-MyHC (22) (cat# M8421, Sigma-Aldrich, Milano, Italy; dilution 1:2000), were detected with proper secondary antibodies (Santa Cruz Biotechnology, Heidelberg, Germany). Samples were counterstained with DAPI, mounted with antifade buffer and then analyzed with confocal microscope Nikon®ECLIPSE 80i (Nikon Instruments S.p.A., Firenze, Italy). For cell-size analysis, myotubes were stained with May-Grünwald Giemsa dye.

For gene expression and/or cell-signaling analysis, differentiated myotubes were harvested by cell scraping, eventually collected with protease inhibitor (PMSF, Sigma Aldrich), pelleted, and stored at −80°C until use.

### Animals

Animal experimentation was conducted in agreement with the National Institutes of Health Principles of Laboratory Animal Care. The study was approved by the local Organization for Animal Welfare with authorization n° 566/2016-PR. Five months old *Rxfp2*^−/−^ male mice (*N* = 12) and wild type control male littermates (*N* = 14) were used. *Rxfp2*^−/−^ mice were generated by mating *Rxfp2*^−/−^ mice and genotyped as previously described (23). Hindlimb denervation was performed on anesthetized animals with ketamine (75 mg/kg) and xylazine (20 mg/kg), by cutting the right hindlimb sciatic nerve and suturing proximal stump into a superficial muscle in order to avoid re-innervation. Animals were sacrificed after 3 or 14 days as described and *Tibialis Anterior, Extensor Digitorum Longus, Soleus*, and *Gastrocnemius* were removed, weighted, and frozen at −80°C until use.

For cross-section area (CSA) analysis, transversal slides of frozen muscles were cut with 10 μm thick and exposed to pre-heated succinate dehydrogenase (SDH) staining solution as previously described (24). Image analysis and cell size measurements were performed with ImageJ® software.

For the analysis of active muscle force, *Extensor Digitorum Longus* and *Soleus* muscles from both denervated and control hindlimbs were individually fixed in a force transducer (KG Scientific Instruments, Heidelberg, Germany) and maintained in oxygenated Krebs solution at the temperature of 25°C. The stimulating conditions were optimized and the length of the muscle was increased until the force development during tetanus was maximal. Cross-sectional areas (calculated from weight and muscle length) were used to normalize muscle force.

The level of testosterone and the weight of seminal vesicles was determined in 4 *Rxfp2*^−/−^ and 5 wild-type control males euthanized at 5 months of age with blood drawn by cardiocentesis. Blood samples were collected at the same time of day, early to mid-morning, in order to rule-out the daily variation of testosterone levels. Serum was collected after centrifugation at 3000 × g for 15 min. The hormone levels were determined in the University of Virginia Center for Research in Reproduction Ligand Assay and Analysis Core (University of Virginia, Charlottesville, VA, USA) using Testosterone ELISA (IBL International, Hamburg, Germany).

### RNA extraction and real time PCR

RNA extraction was performed TRIzol® reagent (Thermofisher) on whole muscle disrupted and homogenized by TissueLyser (Qiagen). RNA was quantified using ND100 NanoDrop. Copy DNA was obtained with SuperScript® III Reverse Transcriptase kit from 400 ng of total RNA. Samples were tested in triplicates for each gene. GAPDH was used as housekeeping gene. Details of forward and reverse primers are reported in Supplemental Table 1. Gene expression data were normalized on wild type-non-denervated samples.

### Protein extraction and western blotting

Protein from cell cultures were extracted by freeze-and-thaw cycles. Muscles were cut into 40 slices, 20 μm thick and lysed with 100 μl of lysis buffer (50 mM Tris pH7.5, 150 mM NaCl, 10 mM MgCl2, 0.5 mM DTT, 1 mM EDTA, 10% glycerol, 2% SDS, 1% Triton from Sigma-Aldrich®, 1:50 Complete Phosphatase inhibitor from Roche®). The total protein concentration was determined using BCA kit (Abcam®, Prodotti Gianni, Milano, Italy). Cell lysates were subjected to SDS-polyacrylamide gel electrophoresis and then western blotting. Primary antibodies were anti-p-AKT (Thr308, cat# 13038), anti-p-mTOR (Ser2448, cat# 2971), anti-p-S6 (Ser 235/236, cat# 4858), monoclonal anti-GAPDH (cat# 5174), anti-LC3 (cat# 4108), anti-P62 (cat# 8025, all from Cell Signaling®; dilution 1:1000), anti-Lys63 (cat# 05-1308), and anti-Lys48 (cat# 05-1307, both from Millipore®; dilution 1:500) (25). Immunoreactive bands were detected using proper HRP-secondary reagent and Clarity Western ECL Substrate (Bio-Rad).

### Statistical analyses

Statistical analysis of the data was conducted with SPSS 21.0 for Windows (SPSS, Chicago, IL). Two-tailed Student's t test was adopted for comparison between two groups, before assessment of normal distribution. Analysis of Variance (ANOVA) with Bonferroni-Holmes correction was adopted for multiple comparisons in cell culture experiments and to compare the effect of treatment (non-denervated, denervated) between the two mouse strains (WT and KO). Error bars in histograms denote mean ± SEM. *P* < 0.05 ware considered to be statistically significant.

## Results

### INSL3/RXFP2 signaling pathway mediates protein synthesis in C2C12 skeletal muscle cell model

**Figure 1:**
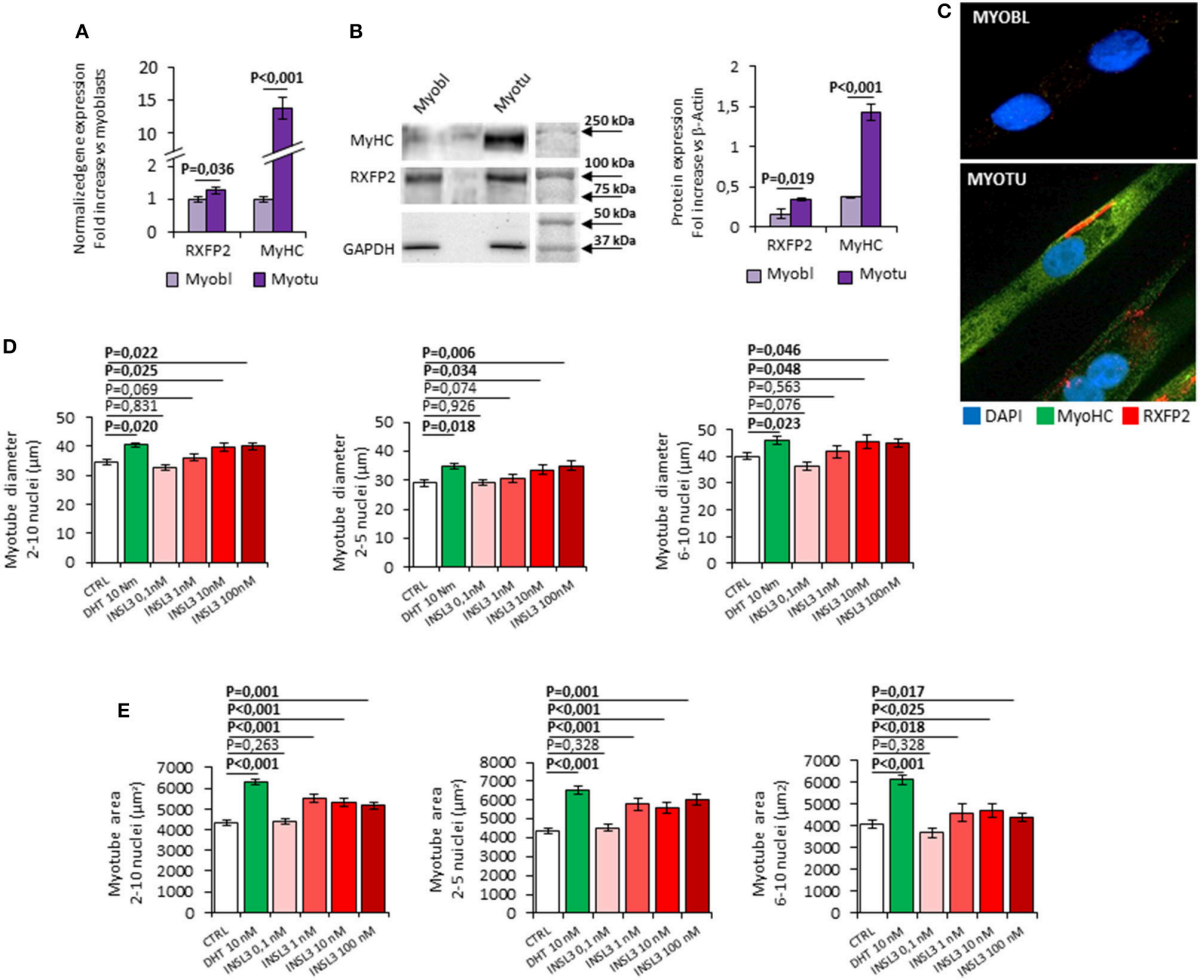
**(A–C)** Expression of RXFP2 and Myosin-Heavy Chain (MyHC) in cultured myoblasts (Myobl) and myotubes (Myotu) from C2C12 mouse cell line. **(A)** Gene expression analysis. Gene expression analyzed using qRT-PCR, normalized to housekeeping β-Actin gene expression, is reported as fold increase vs. expression in myoblasts. **(B)** Representative image of protein expression analysis by western blot and quantification of protein band density. Protein analysis is reported as fold increase vs. GAPDH band density. **(C)** RXFP2 (red) and MyHC (green) staining on myoblasts and myotubes at immunofluorescence. Cells were counterstained with DAPI (blue). **(D,E)** Effect of INSL3 stimulation on mature myotubes cell diameter **(D)** and area **(E)**. Cells were cultured in differentiation medium containing INSL3 at concentration ranging from 0,1 to 100 nM. Dihydrotestosterone (DHT) 10 nM was used as reference trophic factor. Cell size was evaluated in all mature myotubes (2-10 nuclei) and further distinguished into low nuclei- (2-5 nuclei) and high nuclei-clustering (6-10 nuclei). Data are reported as mean values ± standard error of the mean of three independent experiments. Significance: *P*-values between compared conditions are reported. Significant differences (*P* < 0.05) are highlighted in bold.

In order to study the possible role of INSL3/RXFP2 signaling pathway in skeletal muscle, we firstly evaluated the RXFP2 expression and function in the C2C12 muscle cell line (Figure 1). RXFP2 was present at RNA and protein level especially in differentiated multi-nucleated myotubes that express myosin-heavy chain (Figures 1A,B). Confocal microscopy showed that RXFP2 was mainly displayed on cell membrane (Figure 1C). RXFP2 was much less expressed in myoblasts and, accordingly, *in vitro* stimulation of C2C12 myoblasts with INSL3 up to 72h, at concentrations ranging from 0 to 100 nM, had negligible effect on cell proliferation assessed by MTT test (Supplemental Figure 1A).

**Figure 2:**
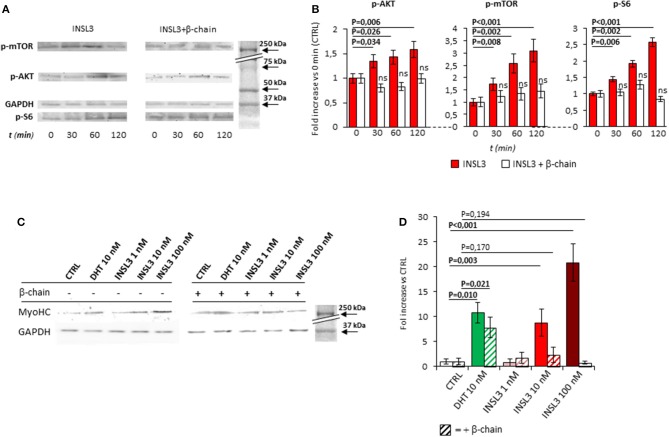
**(A)** Time-course activation of the downstream AKT/mTOR/S6 phosphorylation, in mature myotubes differentiated from C2C12 cells, up-to 120 min after stimulation with INSL3 10 nM, in absence or presence of INSL3-β chain dimer (β-chain, 20 nM) as antagonist of RXFP2 receptor. The signaling pathway activation was monitored by western blot, whose representative results are given. Analysis of band density **(B)**, normalized on GAPDH as loading control, are reported as fold increase compared to basal (CTRL). **(C)** Effect of INSL3 stimulation on Myosin-Heavy Chain (MyHC) protein expression in mature myotubes. C2C12 cells were cultured in differentiation medium containing INSL3 at concentration ranging from 1 to 100 nM, in presence or absence of β-chain. Dihydrotestosterone (DHT) 10 nM was used as reference trophic factor. Protein expression was monitored by western blot, whose representative results are given. Analysis of band density **(D)**, normalized on GAPDH as loading control, are reported as fold increase compared to unstimulated cells (CTRL). Data are reported as mean values ± standard error of the mean of three independent experiments. Significance: *P*-values between compared conditions are reported. Significant differences (*P* < 0.05) are highlighted in bold. Not significant differences in samples co-incubated with INSL3-β chain are marked ns.

We then evaluated the possible effect of RXFP2 stimulation on myotube formation by differentiating C2C12 cells in presence of INSL3, at concentrations ranging from 0 to 100 nM for 7 days. As reference, we treated C2C12 with 10 nM dihydro-testosterone (DHT) to induce hypertrophy (26). None of the different conditions showed a significant effect on either the total number of myotubes or the relative proportion of myotubes with 2-5 nuclei compared to those with 6-10 nuclei (Supplemental Figure 1B). This finding suggests that INSL3 does not affect myoblast differentiation and fusion in the nascent myotube (27). On the other hand, INSL3 treatment resulted in a significant increase of myotubes size when compared to controls (Figures 1D,E). Indeed, myotubes treated with 10 and 100 nM of INSL3 showed an overall increase of the cell diameter, independently of the number of nuclei (Figure 1D). This effect resulted in an overall increase of cell area, in particular for those myotubes with 2-5 nuclei (Figure 1E, *P* < 0,001). Such hypertrophic effect well mimicked the one obtained by DHT, suggesting that INSL3/RXFP2 elicits a growth promoting action similar to DHT. Therefore, we investigated whether Akt/mTOR/S6 signaling, the major pathway that contribute to rates of protein synthesis, is triggered by INSL3 stimulation (28). To this aim, we evaluated downstream Akt/mTOR/S6-phosphorylation at different times after stimulation with 10 nM INSL3 (Figures 2A,B). In order to address the specific involvement of RXFP2 in INSL3-mediated effects, we performed the same experiment in presence of the INSL3-β chain dimer (β-chain, 20 nM), the high affinity antagonist of RXFP2 receptor (19). INSL3 treatment caused a time-dependent increase of both phospho-Akt, phospho-mTOR and phospho-S6 levels. In addition, co-incubation with β-chain did not change the phosphorylation status of the Akt/mTOR/S6 pathway compared to the unstimulated control. Thus, these data indicate the possible influence of the INSL3/RXFP2 signaling on the protein synthesis in myotubes. This hypothesis was ascertained by evaluating the expression of myosin-heavy chain (MyHC) in myotubes, a cell marker of protein synthesis, differentiated in presence of INSL3 at various concentration in absence or presence of β-chain (Figures 2C,D). Western blot analysis revealed that MyHC levels were increased with the higher concentration of INSL3 in the differentiation medium, whilst co-incubation with β-chain significantly blunted this effect. Importantly, incubation with DHT induced a significant increase of MyHC expression even in presence of β-chain, suggesting some degree of independence of the signaling pathways stimulated by the two agonists. Taken together, these results suggest that the INSL3/RXFP2 pathway mediates protein synthesis and hypertrophy in cultured myotubes from C2C12 cell line.

### Rxfp2^−/−^ mice show altered phenotype of β-oxidative fibers at basal condition and after denervation

The possible role of INSL3/RXFP2 pathway on skeletal muscle was assessed *in vivo* through the application of an atrophy model on *Rxfp2*^−/−^ mice (17). To this aim, 5 months old wild-type (WT, *N* = 11) and *Rxfp2*^−/−^ mice (KO, *N* = 9) underwent leg denervation by cutting the sciatic nerve of the left hind limb (DEN), with the right hind limb being used as control (CTRL). The morphological phenotype of muscle fibers, in terms of percentage of glycolytic and β-oxidative fibers and the corresponding CSA, was then evaluated in *tibialis anterior* (*TA*), *gastrocnemius* (*Gastro*), and *soleus* (*Sol*) muscles after 14 days from denervation. Importantly, *Rxfp2* gene was expressed in fast and slow muscles of WT animals, confirmed by of positive immunostaining in cell membrane of WT muscle specimens, and was not induced or suppressed after denervation (Supplemental Figures 1C, D).

**Figure 3:**
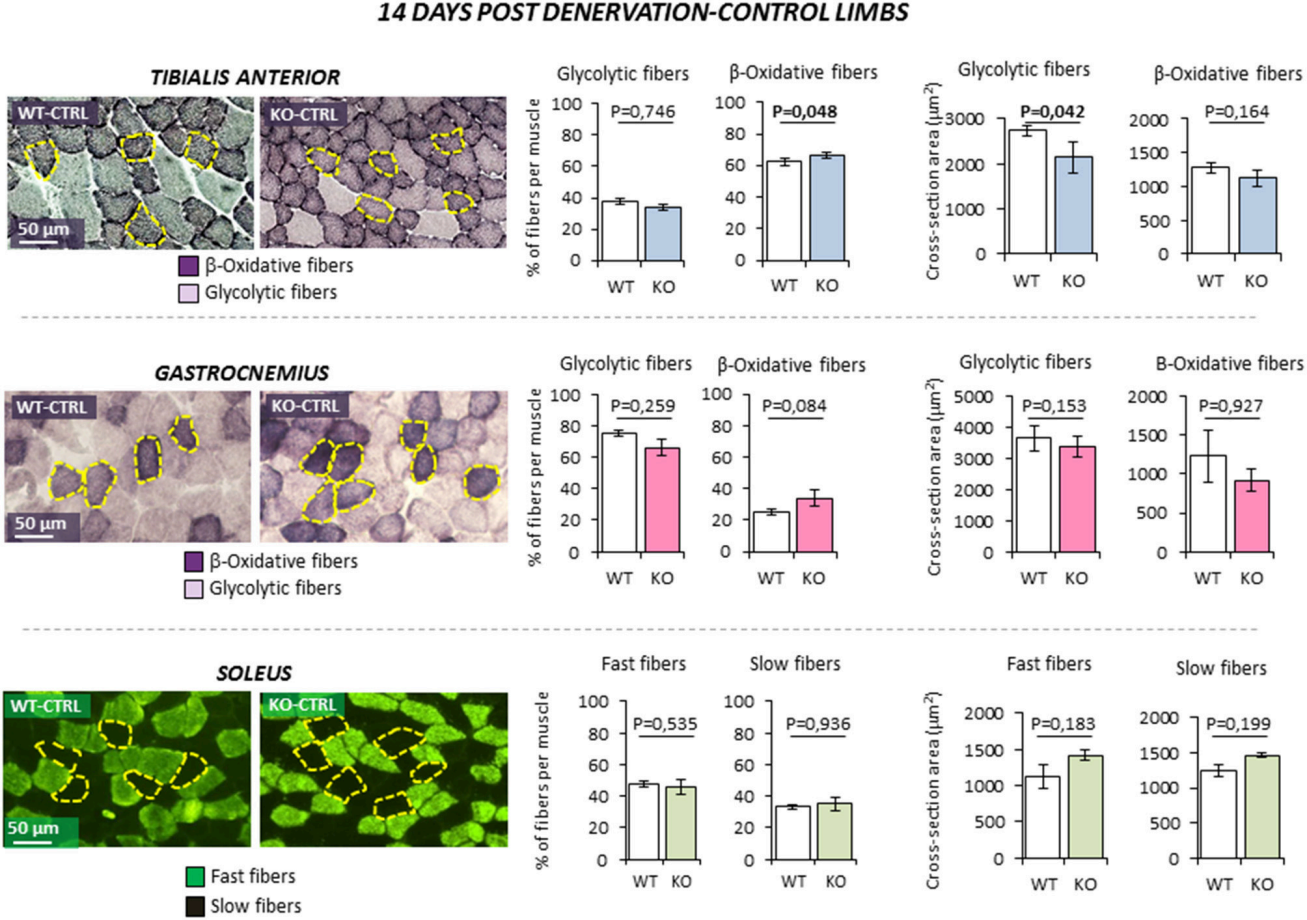
Effect of leg denervation on fiber composition and cross section area in *tibialis anterior, gastrocnemius*, and *soleus* muscles in wild type (WT, *N* = 6) and *Rxfp2*^−/−^ mice (OK, *N* = 5). Results obtained in contralateral non-denervated control limbs (CTRL) are given after 14 days from denervation. Data in *tibialis anterior, gastrocnemius* muscle specimens were obtained by succinate-dehydrogenase (SDH) staining, distinguishing β-oxidative (purple) and glycolytic fibers (pale). Data in *soleus* muscle specimens were obtained by myosin-heavy chain immune-staining, distinguishing fast (green), and glycolytic fibers (dark). Representative microscopic fields are given. Results on fiber composition and cross section area are reported respectively as percentage of fiber-type per muscle and absolute fiber area (μm^2^).Data are reported as mean values ± standard error of the mean. Significance: *P*-values between compared conditions are reported. Significant differences (*P* < 0.05) are highlighted in bold.

In control innervated limbs (Figure 3), *TA* from KO animals showed a slight increase in the percentage of β-oxidative fibers (*P* = 0.048). Interestingly, CSA of glycolytic fibers was significantly reduced when compared to WT (*P* = 0.042). No significant difference in terms of fiber type or fiber size was observed in *Gastro* and *Sol* from KO mice in basal condition.

**Figure 4:**
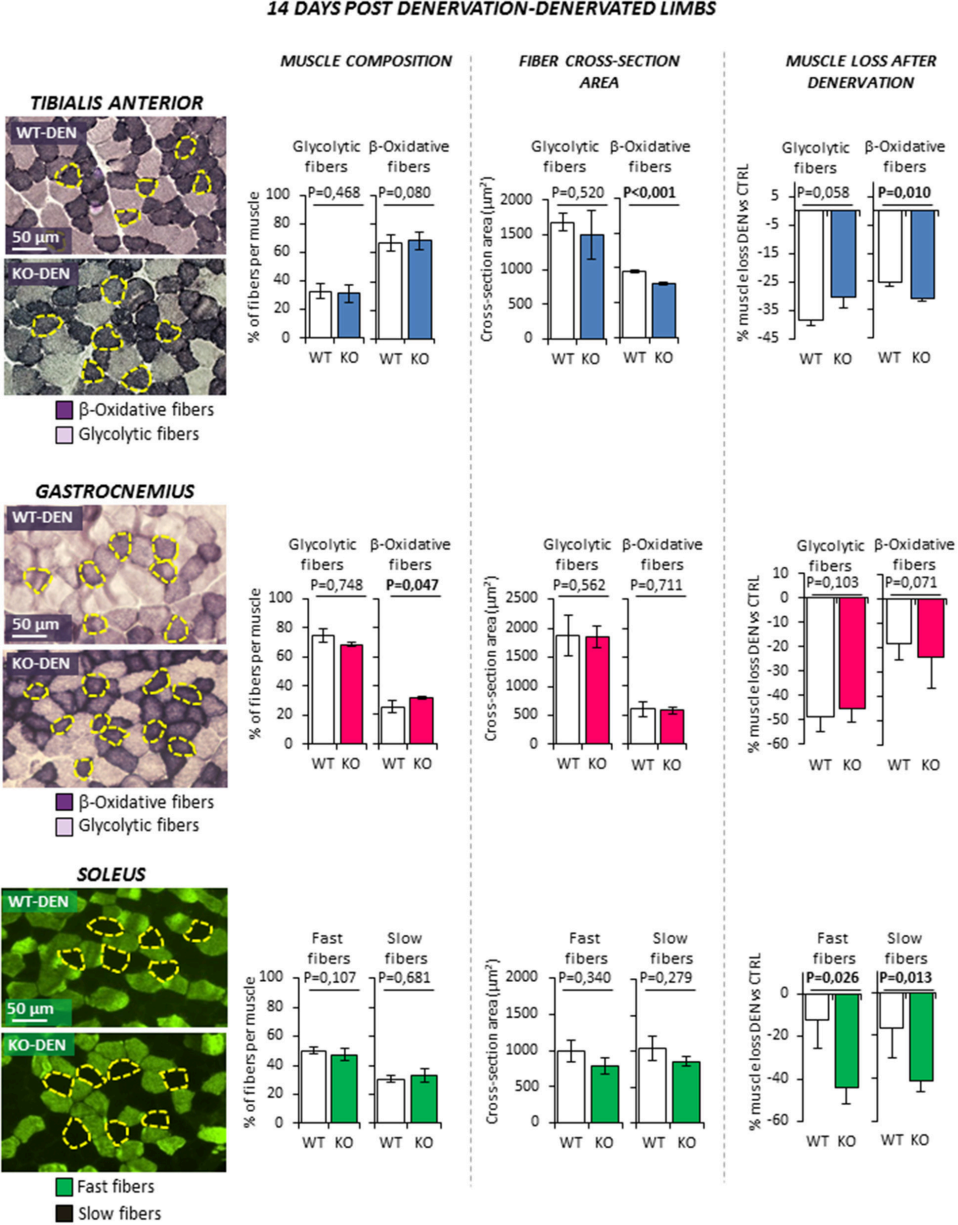
Effect of leg denervation on fiber composition and cross section area in *tibialis anterior, gastrocnemius* and *soleus* muscles in wild type (WT, *N* = 6) and *Rxfp2*^−/−^ mice (KO, *N* = 5). Results obtained in denervated limbs (DEN) are given after 14 days from denervation. Data in *tibialis anterior, gastrocnemius* muscle specimens were obtained by succinate-dehydrogenase (SDH) staining, distinguishing β-oxidative (purple), and glycolytic fibers (pale). Data in soleus muscle specimens were obtained by myosin-heavy chain immune-staining, distinguishing fast (green), and glycolytic fibers (dark). Representative microscopic fields are given. Results on fiber composition and cross section area are reported respectively as percentage of fiber-type per muscle and absolute fiber area (μm^2^). Data on muscle loss after denervation were obtained as percentage of cross section area reduction in denervated (DEN) vs. control (CTRL) limbs.Data are reported as mean values ± standard error of the mean. Significance: *P*-values between compared conditions are reported. Significant differences (*P* < 0.05) are highlighted in bold.

Denervation (Figure 4) was associated to a significant reduction of CSA of both fiber types in *TA* muscle as expected (all *P* < 0.01. Data not shown). However, β-oxidative fibers of KO animal showed a further reduction of CSA compared to WT littermates, resulting in a greater muscle loss (−25.4 ± 1.1% WT vs. −31.0 ± 1.0% KO; *P* = 0.010). Similarly, denervation induced the reduction of CSA of both fiber types in *Gastro* (all *P* < 0.01. Data not shown) and a slight increase of the percentage of β-oxidative fibers (*P* < 0.047), but no significant difference was observed between WT and KO mice. On the other hand, *Sol* muscle showed the most peculiar pattern. Indeed, because *Sol* contains essentially fast-twitch oxidative 2A (fast fatigue resistant fibers) and slow-twitch type 1 myosins (slow fibers), it differs from the former two fast muscles. Denervation was not associated with a significant variation of fiber type composition in WT and KO animals. Interestingly, KO mice showed an enhanced muscle loss after denervation when compared to controls. Indeed, the percentage of decrease in CSA after denervation was significantly higher in fast and slow fibers when compared to controls (fast fibers −12.1 ± 13.6% WT vs. −44.0 ± 7.9% KO, *P* = 0.026; slow fibers −16.7 ± 13.7% WT vs.−41.5 ± 4.5% KO, *P* = 0.013).

Taken together, these data suggest that *RXFP2* plays an important trophic action in β-oxidative muscle fibers. This effect is particularly evident in slow muscles such as *Sol* but also in the β-oxidative portion of fast muscles such as *TA*.

### Rxfp2^−/−^ mice show alteration in the signaling pathway that controls protein-synthesis after denervation

In order to address the molecular mechanisms underlying the observed effect of *Rxfp2* ablation *in vivo*, we evaluated the major signaling pathways involved in protein turnover and muscle mass maintenance.

Atrophy triggers a complex cascade of intracellular events that culminates with the activation of a transcriptional-dependent program for the expression of the atrophy-related genes or “atrogenes” (29). Therefore, we checked the expression of several atrophy-related ubiquitin-ligases such as *Atrogin-1, MuRF-1, MUSA1, SMART, FBXO31*, and *TRAF6*, in *TA* and *Sol*. In particular, we examined muscles that were in early stage- (3 days) and late stage (14 days) of the atrophy process.

**Figure 5:**
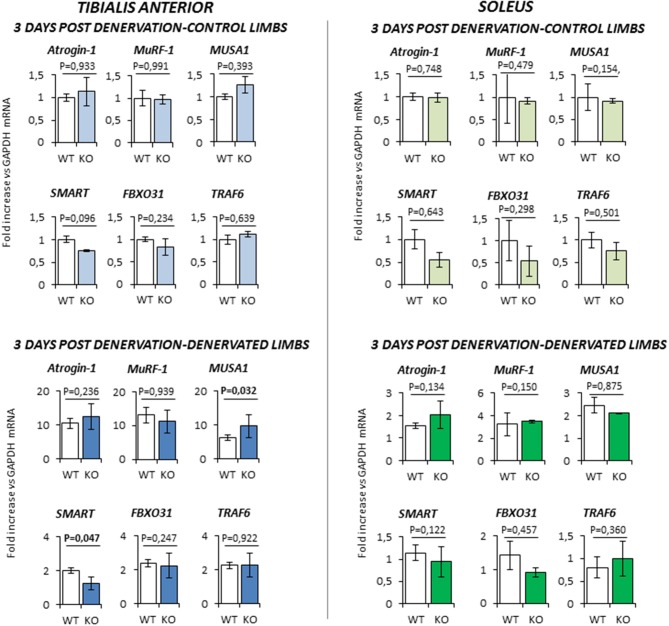
Gene expression analysis in both contralateral control limbs and denervated limbs in wild type (WT, *N* = 5) and *Rxfp2*^−/−^ mice (KO, *N* = 4), 3 days after denervation. Data in tibialis anterior and soleus muscle specimens are reported for *Atrogin-1, MuRF-1, MUSA1, SMART, FBOX31*, and *TRAF6* genes, whose expression is normalized on GAPDH as housekeeping. Data are reported as mean values ± standard error of the mean. Significance: *P*-values between compared conditions are reported. Significant differences (*P* < 0.05) are highlighted in bold.

At 3 days-post denervation (Figure 5), control limbs from either WT and KO animals showed no differential expression of these ubiquitin ligases in both *TA* and *Sol*. However, TA showed a significant up-regulation of *MUSA1* and a downregulation of *SMART* (*P* = 0.032 and *P* = 0.047, respectively) in KO mice.

**Figure 6:**
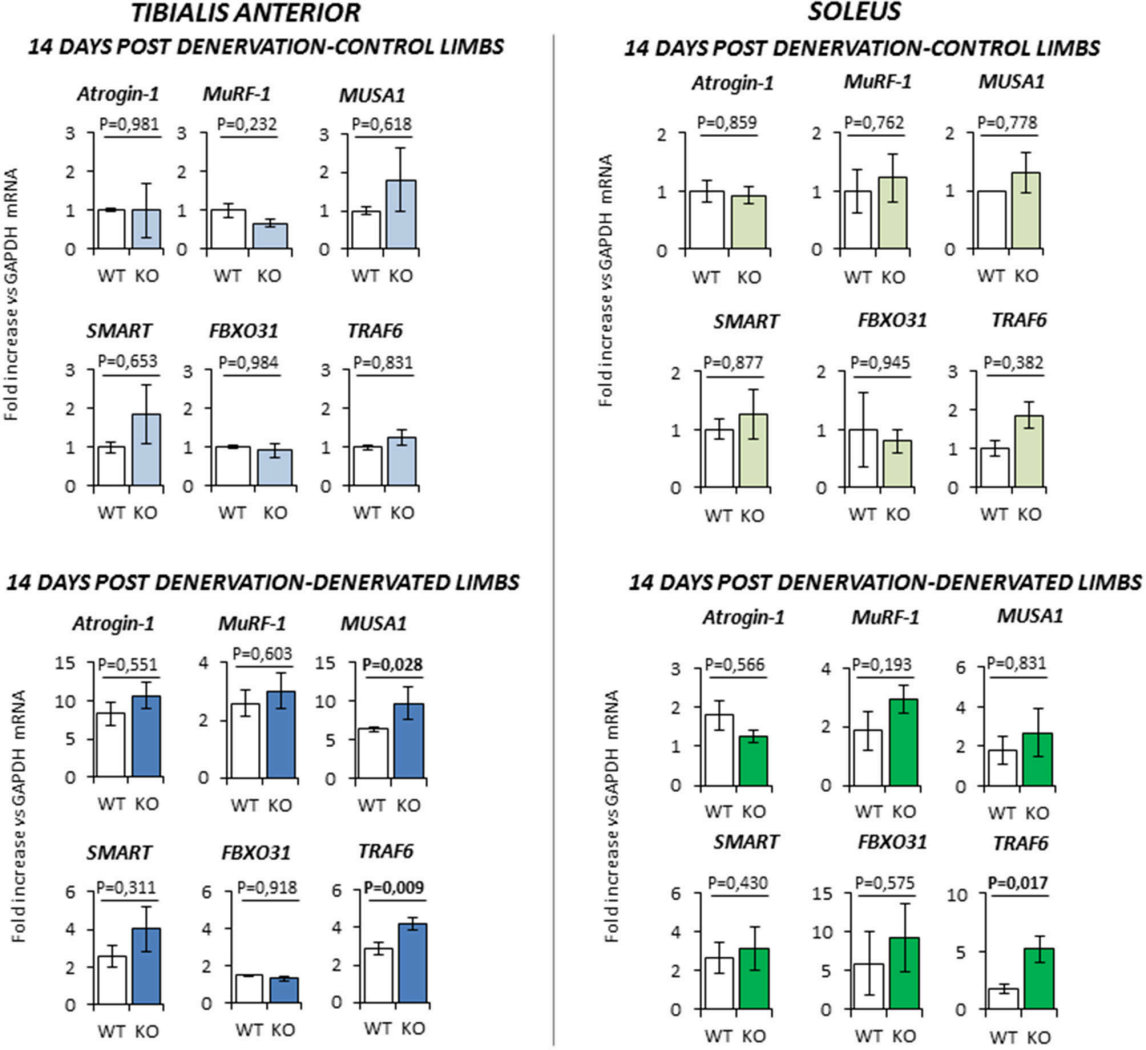
Gene expression analysis in both contralateral control limbs and denervated limbs in wild type (WT, *N* = 6) and *Rxfp2*^−/−^ mice (KO, *N* = 5), 14 days after denervation. Data in tibialis anterior and soleus muscle specimens are reported for *Atrogin-1, MuRF-1, MUSA1, SMART, FBOX31*, and *TRAF6* genes, whose expression is normalized on GAPDH as housekeeping. Data are reported as mean values ± standard error of the mean. Significance: *P*-values between compared conditions are reported. Significant differences (*P* < 0.05) are highlighted in bold.

At 14 days-post denervation (Figure 6), despite innervated limbs of controls and KO mice show a similar level of expression of these genes, denervated *TA* and *Sol* of KO animals displayed a more robust induction of *TRAF6* (respectively *P* = 0.009 and *P* = 0.017 vs. WT) whilst TA showed also a significant up-regulation of *MUSA1* (*P* = 0.028 vs. WT).

**Figure 7:**
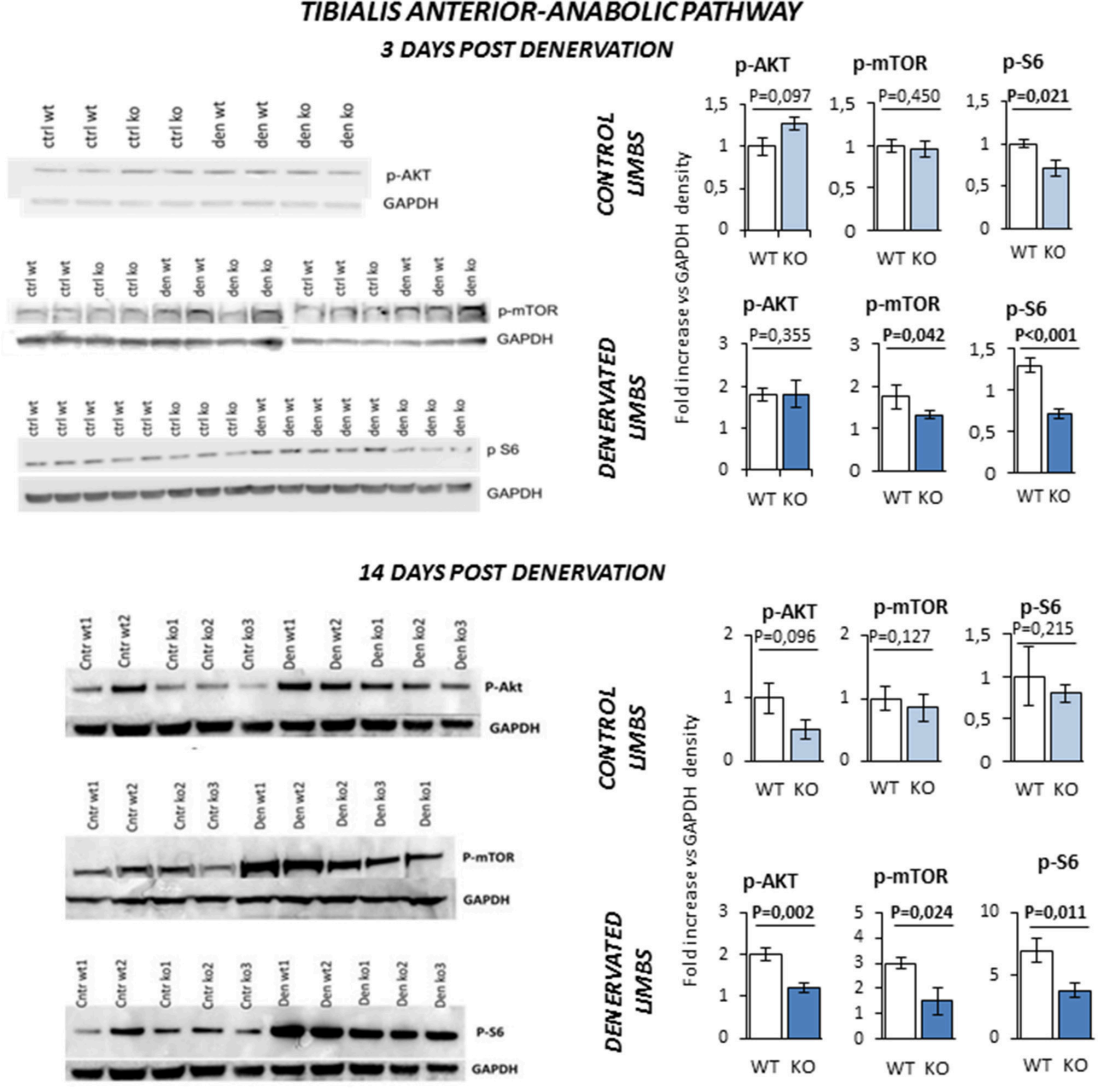
Activation of anabolic pathway in *tibialis anterior* muscle specimens obtained from contralateral non-denervated (CONTROL) and denervated (DENERVATED) limbs in wild type (WT) and *Rxfp2*^−/−^ mice (KO), at both 3 (WT, *N* = 5; KO, *N* = 4) and 14 (WT, *N* = 6; KO, *N* = 5) days post-denervation. Downstream phosphorylation of AKT/mTOR/S6 pathway was monitored by western blot analysis, whose representative images are given. Target band densities were normalized on corresponding GAPDH as loading control. Data are reported as mean values ± standard error of the mean. Significance: *P*-values between compared conditions are reported. Significant differences (*P* < 0.05) are highlighted in bold.

Next, we monitored the status of the Akt/mTOR/S6 signaling, the major pathway that controls protein synthesis in myofibers. *TA* of KO mice was more prone to muscle loss, like *Sol*, and showed a pattern of expression of atrophy-related genes that was similar to *Sol*. Since *TA* has a much more muscle mass than *Sol*, we used this muscle for the biochemical analyses of the signaling pathways controlling protein synthesis and degradation. *TA* from innervated limbs of KO and WT did not differ in terms of phosphorylation status of Akt and mTOR (Figure 7). However, p-S6 was significantly lower in the control innervated leg of KO mice compared to WT at 3 days. After denervation, TA of WT animals showed a global compensatory activation of the Akt/mTOR/S6 pathway as previously reported (24). However, a significant impairment of this compensatory stimulation was observed in KO mice at both 3 and 14 days after denervation. In fact, KO mice showed a significant reduction of phospho-Akt, phospho-mTOR and phospho-S6 levels when compared to controls.

**Figure 8:**
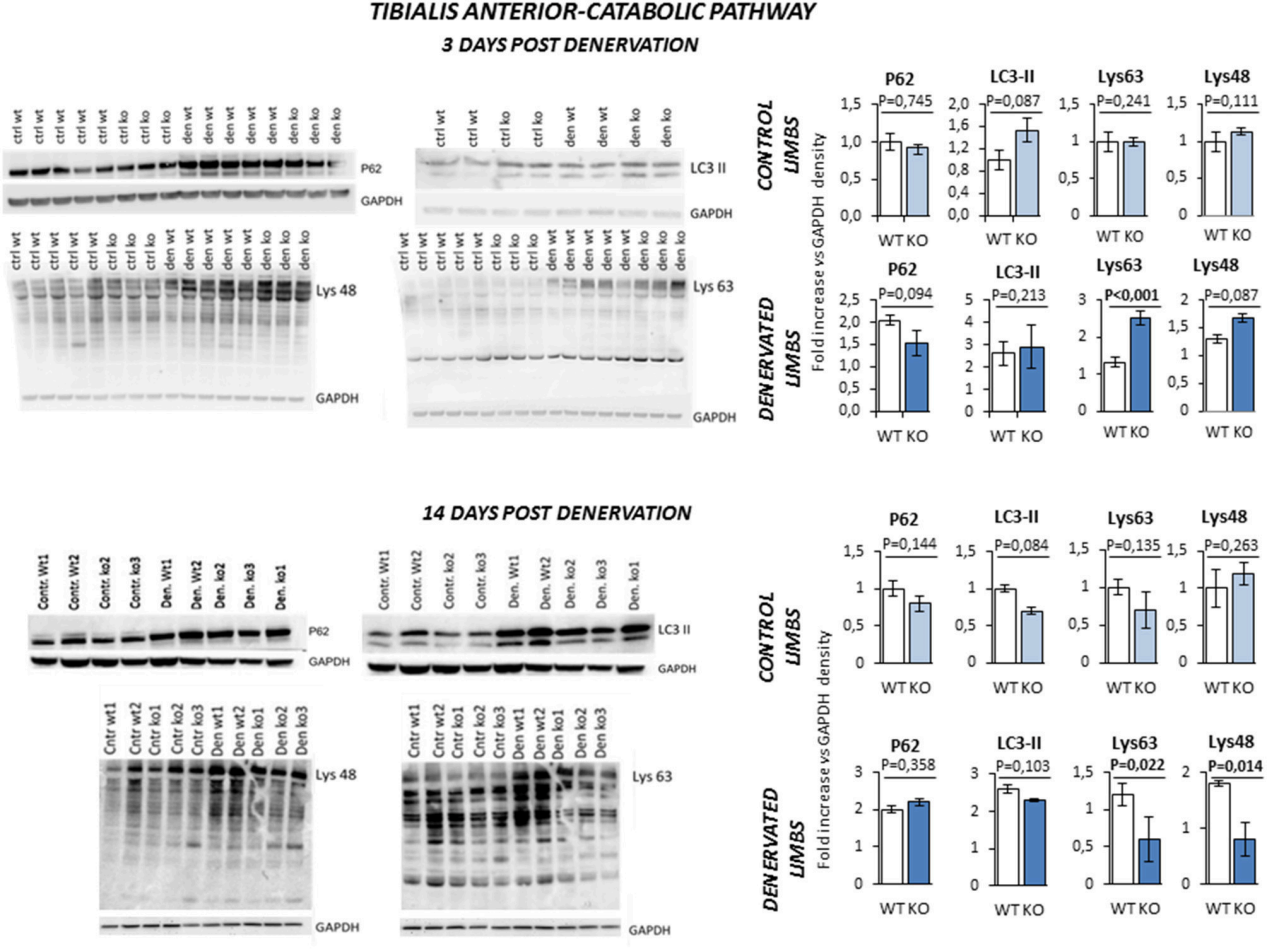
Activation of catabolic pathway in *tibialis anterior* muscle specimens obtained from contralateral non-denervated (CONTROL) and denervated (DENERVATED) limbs in wild type (WT) and *Rxfp2*^−/−^ mice (KO), at both 3 (WT, *N* = 5; KO, *N* = 4) and 14 (WT, *N* = 6; KO, *N* = 5) days post-denervation. LC3-II and P62, markers of cell autophagy, and Lys-48 and Lys-63 poly-ubiquitination (poly-U), markers of the activation of the proteasome system, were monitored by western blot analysis, whose representative images are given. Target band densities were normalized on corresponding GAPDH as loading control. Data are reported as mean values ± standard error of the mean. Significance: *P*-values between compared conditions are reported. Significant differences (*P* < 0.05) are highlighted in bold.

Next, we evaluated the pathways that control protein breakdown (Figure 8). Protein degradation is mainly controlled by the autophagy and the ubiquitin-proteasome system (2). Any unbalance of the autophagy process was ruled out by the evidence that LC3-II and P62 were equally upregulated in WT and KO mice after 3 and 14 days of denervation. On the other hand, Lys-48 and Lys-63 poly-ubiquitinated proteins (poly-U), a readout of the activation of the ubiquitin-proteasome system, showed a peculiar time- and genotype-dependent pattern. KO mice showed higher levels of poly-U Lys-48 and Lys-63 than controls after 3 days of denervation. However, this pattern was reverted after 14 days of denervation suggesting a transient activation of the proteasome that degrades the proteins that were ubiquitinated by the E3 ligases.

It should be noted that the expression of the androgen receptor gene was unaffected by the ablation of *Rxfp2* gene or denervation (Supplemental Figure 1E) in agreement with previous reports (30). Furthermore, KO and WT animals did not differ in terms of serum T levels (respectively, 44.9 ± 6.1 ng/dL vs. 45.1 ± 3.2 ng/dL; *P* = 0,575) and weight of seminal vesicles (0.152 ± 0.015 gr vs. 0.195 ± 0.019 gr; *P* = 0.321), a sensible marker of androgen production and signaling. This data ruled out any compensatory effect of the androgen signaling in the KO mice.

Taken together, these data suggest that INSL3/RXFP2 signaling sustains the compensatory protein synthesis program that takes place in skeletal muscle in course of atrophy and concomitantly negatively regulates the ubiquitination process and the proteasome activity.

### Soleus muscles in RXFP2^−/−^ mice show reduced force generation after denervation

**Figure 9:**
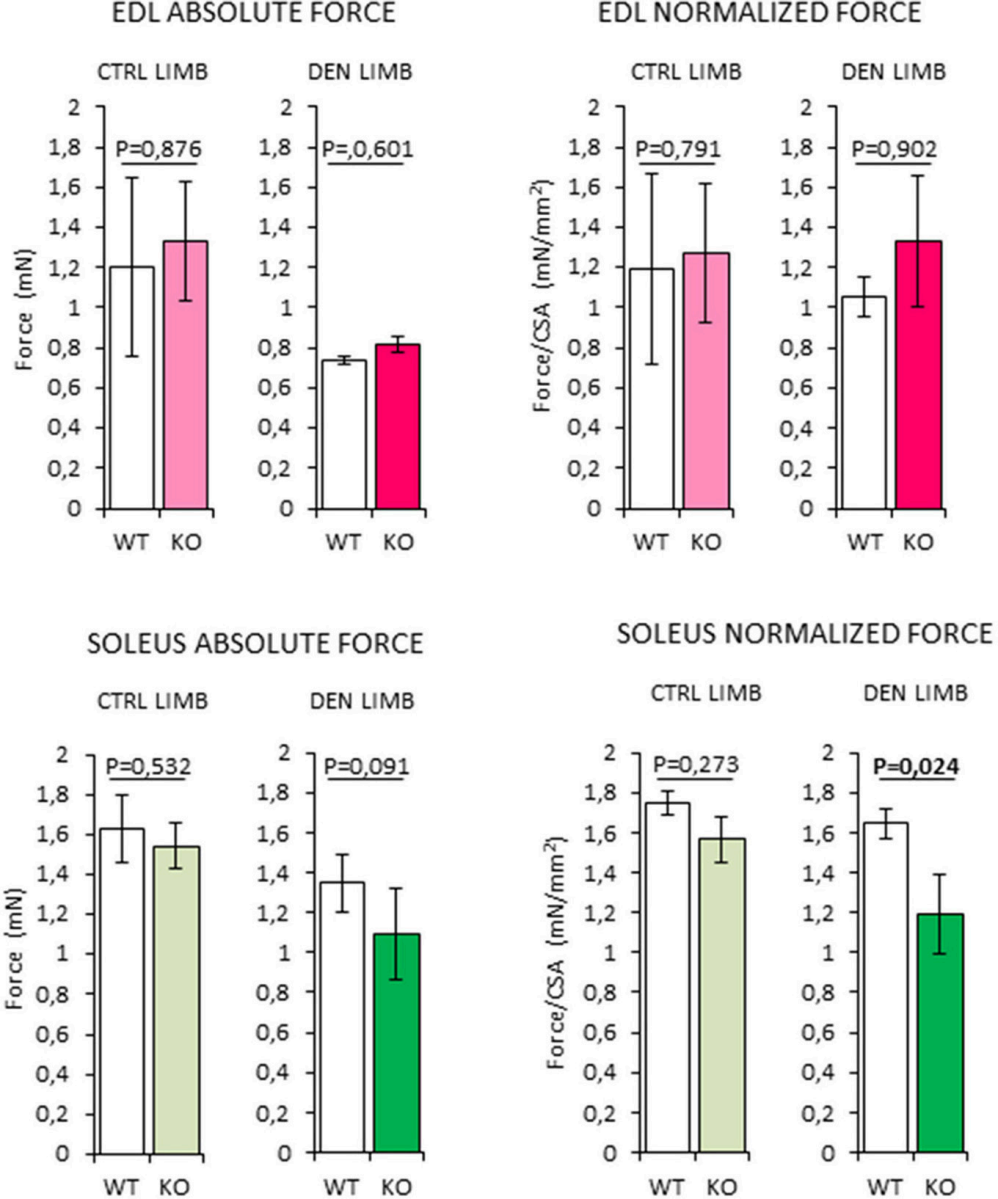
Analysis of absolute and normalized force at tetanic contraction in excised *extensor digitorum longus* (*EDL*) and *SOLEUS* muscles, from contralateral non-denervated (CTRL) and denervated (DEN) limbs, in wild type (WT, *N* = 3) and *Rxfp2*^−/−^ mice (KO, *N* = 3). Data are reported as mean values ± standard error of the mean. Significance: *P*-values between compared conditions are reported. Significant differences (*P* < 0.05) are highlighted in bold.

In order to assess whether the changes in muscle morphology in *Rxfp2*^−/−^ mice were accompanied by alteration of muscle strength, absolute and normalized force at tetanic contraction was measured in *extensor digitorum longus* (*EDL*) and *Sol* muscles (Figure 9). Denervation caused reduction of absolute force of *EDL* from WT and KO mice. When force was normalized to muscle mass (Supplemental Figure 1F), there was no difference between innervated and denervated muscles and between the different genotypes.

On the other hand, denervated *Sol* muscles from the KO mice showed a trend toward a reduction in absolute force that became significant when force was normalized for muscle mass (*P* = 0.024 vs. WT).

Taken together, these data suggest that the impairment of INSL3/RXFP2 signaling is associated with a reduction of the intrinsic contractile function skeletal muscles with higher oxidative metabolism.

## Discussion

In this study, through a combined *in vitro* and *in vivo* approach, we provide evidence for the first time that the testicular peptide hormone INSL3 plays a role in skeletal muscle metabolism and function. We showed that INSL3 exerts a trophic effect on differentiated C2C12 myotubes as it stimulates cell size and protein synthesis through the RXFP2 mediated pathway. The experiments with an *in vivo* animal model, the *Rxfp2*^−/−^ mice, confirmed that INSL3/RXFP2 signaling affects protein turnover in beta oxidative fibers. From a mechanistic point of view, the ablation of INSL3/RXFP2 signaling was associated with an alteration of the ubiquitin-proteasome system and a concomitant impairment in the anabolic Akt pathway, activated to counteract muscle loss in order to sustain the metabolic changes of atrophying muscles and to maintain the myofiber (2). Altogether, the abnormal regulation of protein turnover leads to an exacerbated muscle loss in KO mice. Most importantly, these molecular data had a functional counterpart since a reduced contractile force was observed in muscles with a highly β-oxidative metabolism like *Soleus*. Thus, it can be hypothesized that INSL3/RXFP2 signaling displays an overall anabolic role on skeletal muscle, preserving from protein loss (31). The fact that some muscles are more affected than the others is not surprising. A new concept is emerging from the literature that considers the atrophy program specific for each muscle. We and others have found that in the same catabolic condition each muscle respond differently in terms of muscle loss, atrogene induction, and activation of the degradation systems and signaling pathways that control protein turnover (32).

In last years, accumulating evidence suggests that T and INSL3 display a large degree of overlap. In fact, both hormones are produced by the Leydig cells and their serum levels are strictly correlated with the muscle mass profile in humans during lifetime, achieving a post-pubertal peak and a progressive decline during aging (33). Furthermore, INSL3 was shown to have an anabolic role on bone, a typical target tissue of T (17), and in turn T is a well-known hormone with trophic effects in skeletal muscle (34). In this context, our results extend and support the view that INSL3 plays an important role in in extra-genital tissues and that the testicular hormones have a joined anabolic effect on the bone-muscle unit (10, 35). From a physiological perspective, such a redundancy of trophic factors is not surprising, being frequently observed for target tissues subjected to mechanical stress like bone and skeletal muscle itself (36–39).

In addition, our data underlie some important pathophysiological and clinical cues. Indeed, sarcopenia, together with osteoporosis and other symptoms, is among the most diffuse condition associated with clinical hypogonadism that manifests with the reduced production of both T and INSL3 (11, 12, 40). However, in cases of milder testicular abnormalities known as subclinical hypogonadism, there are no apparent variation of T levels but low serum levels of INSL3 are present. In such patients, muscle fatigue is equally associated with the loss of bone and muscle mass (6, 41). On the other hand, known inactivating mutations of *Rxfp2* gene in humans are associated with altered bone metabolism with no significant variation of androgen pattern (17). Therefore, it is clearly emerging that reduction in serum levels or function of INSL3 could contribute to signs and symptoms of male hypogonadism, alone or in combination with T deficiency. It should be noted that within the spectrum on muscle fiber types observed in mammalians, ranging from slow oxidative fast-twitch oxidative glycolytic (2A), fast-twitch glycolytic fibers (2B) and type 2X fibers with intermediate fatigue resistance, 2B fibers are not detectable in humans (42). In light that major effects of *Rxfp2* ablation have been observed in fibers with β-oxidative metabolism, and given the peculiar composition of human skeletal muscles richer in fibers with a *Soleus*-like metabolism, INSL3/RXFP2-signaling is expected to exert a wider effect in human than in mouse model.

We can clearly account for some drawbacks in this study. First, the chemical stability of INSL3 was not evaluated for *in vitro* experiments. Indeed, previous reports showed some sensitivity of INSL3 to insulin-degrading enzyme, although with a much lower extent compared to insulin (43). Interestingly, available studies on cell signaling performed acute stimulation with mouse INSL3 up to 18 h, documenting no significant impairment of the experimental model (44). Considering the low concentration of horse serum used *in vitro* and the frequent renewal of culture medium containing the agonist, major effects due to degradation of INSL3 can be reasonably ruled out. Second, relaxin is produced by the prostate gland in males and displays a three orders of magnitude lower affinity for RXFP2 compared to INSL3 (9), thus a possible productive binding of this peptide to muscle RXFP2 cannot be excluded. However, we found that WT and KO mice were comparable in terms of androgen production and development of androgen responsive organs, such as seminal vescicles. We can thus suppose an overlapping prostate function between the two mice stains, supporting no differential contribution of the relaxin effect on skeletal muscle, if ever. Third, in this preliminary report we were not able to provide a similar correlation in humans, and we did not assess directly the possible stimulating effect of exogenous INSL3 on muscle phenotype, clarifying the aforementioned limitations. Further studies are warranted to address these questions.

## Conclusions

Here we provide evidence for the first time that the ablation of INSL3/RXFP2-signaling has detrimental effect on skeletal muscle, showing that *Rxfp2*^−/−^ mice display worsened muscle loss and contractile force reduction after denervation compared to wild type controls, particularly in muscles with a highly β-oxidative metabolism. Mechanistically, a major involvement of the alteration of the ubiquitin-proteasome system is suggested. This pattern is expected to be strengthened in humans, according to both the peculiar metabolic characteristics of muscle fibers and the endocrine dynamic of INSL3. Further investigations are warranted to address these insights.

## Author contributions

AF, MS, and LDT conceived and designed the project; GL, SB, AAr, and BB performed the experiments with assistance from AF, LDT, and BB. AAg, GL, BB, and LDT analyzed the data. AAg contributed the *Rxfp*^2−/−^ mouse model; AAg and CF provided intellectual contribution; AF, LDT, and MS wrote the manuscript.

### Conflict of interest statement

The authors declare that the research was conducted in the absence of any commercial or financial relationships that could be construed as a potential conflict of interest.

## Supplementary material

Supplemental Figure 1 **(A)** Effect on C2C12 cell proliferation of INSL3 stimulation for 24–72 h, monitored by MTT assay. INSL3 was used at concentration ranging from 0,1 to 100 nM. Dihydrotestosterone (DHT) 10 nM was used as reference trophic factor. Results are reported as optical density (Abs.) at 595 nm of cell lysates. Data are reported as mean values ± standard error of the mean and are representative of three independent experiments. **(B)** Effect of INSL3 stimulation on the number of myotubes differentiated from C2C12 cells. Data are reported as total number of myotubes per well, further distinguished as percentage of myotubes with low nuclei- (2-5 nuclei) and high nuclei-clustering (6-10). Data are reported as mean values ± standard error of the mean and are representative of three independent experiments. **(C)** *Rxfp2* gene expression analysis in muscle specimens, from both contralateral non-denervated (CTRL) and denervated limbs, of wild type animals (*N* = 3). Normalized expression on *GAPDH* is reported as fold increase vs. corresponding CTRL. Data are reported as mean values ± standard error of the mean. **(D)** Representative immunostainings for RXFP2 (red) and myosin heavy chain (MyHC, green) in muscle fibers from *Gastrocnemius* specimens obtained from wild type (WT) and *Rxfp2*^−/−^ (KO) mice. Samples were counterstained with 4′,6-Diamidine-2′-phenylindole (DAPI, blue). **(E)** Androgen receptor (AR) gene expression analysis in muscle specimens, from both contralateral non-denervated (CTRL) and denervated limbs (DEN), of wild type (WT, *N* = 3) and *Rxfp2*^−/−^ mice (KO, *N* = 3). Data of gastrocnemius (*Gastro*), tibialis anterior (*TA*), and soleus (*Sol*) are reported. *AR* expression is normalized on GAPDH as housekeeping. Data are reported as mean values ± standard error of the mean. **(F)** *Extensor digitorum longus* (*EDL*) and *soleus* cross section area (CSA) in the evaluation of contraction force for wild type (WT, *N* = 3) and *RXFP2*^−/−^ mice (KO, *N* = 3). CSA was calculated by dividing the wet muscle weight for optimal muscle length. Data of denervated limbs (DEN) was normalized on contralateral non-denervated specimens (CTRL). Data are reported as mean values ± standard error of the mean.

Supplemental Table 1 Primers used for quantitative PCR Analyses.

## Abbreviations

INSL3: Insulin-like Peptide 3
β-chain: INSL3-β chain dimer
MyHC: Myosin Heavy Chain
CSA: cross-section area
*TA*: *tibialis anterior*
*Gastro*: *gastrocnemius*
*Sol*: *Soleus*
*EDL*: *extensor digitorum longus*
T: testosterone
DHT: dihydrotestosterone.
