# Supplementary figures and images for: Protective Role of Testicular Hormone INSL3 From Atrophy and Weakness in Skeletal Muscle
Source: Front Endocrinol (Lausanne). 2018 Sep 28;9:562. doi: 10.3389/fendo.2018.00562 (PMC6172310; doi:10.3389/fendo.2018.00562)

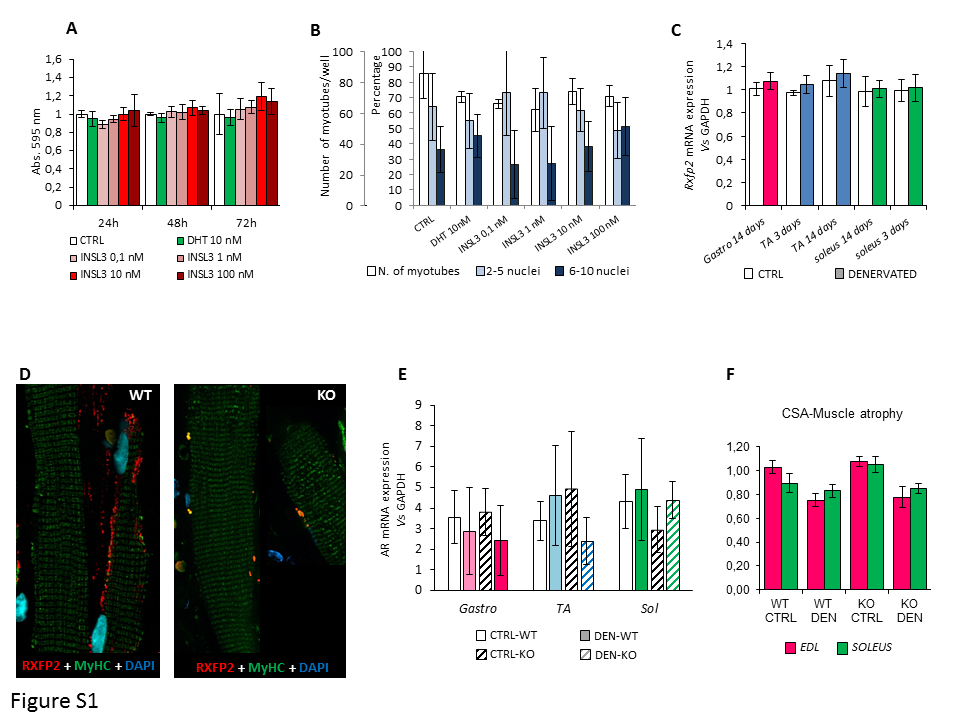

Supplement: Supplemental Figure 1 — (A) Effect on C2C12 cell proliferation of INSL3 stimulation for 24–72 h, monitored by MTT assay. INSL3 was used at concentration ranging from 0,1 to 100 nM. Dihydrotestosterone (DHT) 10 nM was used as reference trophic factor. Results are reported as optical density (Abs.) at 595 nm of cell lysates. Data are reported as mean values ± standard error of the mean and are representative of three independent experiments. (B) Effect of INSL3 stimulation on the number of myotubes differentiated from C2C12 cells. Data are reported as total number of myotubes per well, further distinguished as percentage of myotubes with low nuclei- (2-5 nuclei) and high nuclei-clustering (6-10). Data are reported as mean values ± standard error of the mean and are representative of three independent experiments. (C) Rxfp2 gene expression analysis in muscle specimens, from both contralateral non-denervated (CTRL) and denervated limbs, of wild type animals (N = 3). Normalized expression on GAPDH is reported as fold increase vs. corresponding CTRL. Data are reported as mean values ± standard error of the mean. (D) Representative immunostainings for RXFP2 (red) and myosin heavy chain (MyHC, green) in muscle fibers from Gastrocnemius specimens obtained from wild type (WT) and Rxfp2−/− (KO) mice. Samples were counterstained with 4′,6-Diamidine-2′-phenylindole (DAPI, blue). (E) Androgen receptor (AR) gene expression analysis in muscle specimens, from both contralateral non-denervated (CTRL) and denervated limbs (DEN), of wild type (WT, N = 3) and Rxfp2−/− mice (KO, N = 3). Data of gastrocnemius (Gastro), tibialis anterior (TA), and soleus (Sol) are reported. AR expression is normalized on GAPDH as housekeeping. Data are reported as mean values ± standard error of the mean. (F) Extensor digitorum longus (EDL) and soleus cross section area (CSA) in the evaluation of contraction force for wild type (WT, N = 3) and RXFP2−/− mice (KO, N = 3). CSA was calculated by dividing the wet muscle weight for opt [file Image_1.TIF]
