# Supplementary material for: Protective Role of Testicular Hormone INSL3 From Atrophy and Weakness in Skeletal Muscle
Source: Front Endocrinol (Lausanne). 2018 Sep 28;9:562. doi: 10.3389/fendo.2018.00562 (PMC6172310; doi:10.3389/fendo.2018.00562)
Supplement: Supplemental Table 1 — Primers used for quantitative PCR Analyses. [file Table_1.DOCX]

**Supplemental Table 1**

Primers used for quantitative PCR Analyses

| GENE | Forward primer (5’-3’) | Reverse primer (5’-3’) |
| --- | --- | --- |
| Atrogin-1 | GCAAACACTGCCACATTCTCTC | CTTGAGGGGAAAGTGAGACG |
| SMART | TCAATAACCTCAAGGCGTTC | GTTTTGCACACAAGCTCCA |
| FBXO31 | GTATGGCGTTTGTGAGAACC | AGCCCCAAAATGTGTCTGTA |
| MUSA-1 | TCGTGGAATGGTAATCTTGC | CCTCCCGTTTCTCTATCACG |
| MuRF-1 | ACCTGCTGGTGGAAAACATC | ACCTGCTGGTGGAAAACATC |
| TRAF6 | GCAGTGAAAGATGACAGCGTGA | TCCCGTAAAGCCATCAAGCA |
| GAPDH | CACCATCTTCCAGGAGCGAG | CCTTCTCCATGGTGGTGAAGAC |
